# Organ Donor Transmission of *Rickettsia typhi* to Kidney Transplant Recipients, Texas, USA, 2024

**DOI:** 10.3201/eid3110.250961

**Published:** 2025-10

**Authors:** Jeffrey C. Jones, Omar G. García, Julian A. Villalba, Rosa Hinojosa, Marissa L. Taylor, Pallavi Annambhotla, Matthias H. Kapturczak, Bonny Mayes, Sandor E. Karpathy, Arlyn N. Gleaton, Linda Moon, Joseph Singleton, Sridhar V. Basavaraju, Christopher D. Paddock

**Affiliations:** San Antonio Infectious Diseases Consultants, San Antonio, Texas, USA (J.C. Jones); City of Laredo Public Health Department, Laredo, Texas, USA (O.G. García); Centers for Disease Control and Prevention, Atlanta, Georgia, USA (J.A. Villalba, M.L. Taylor, P. Annambhotla, S.E. Karpathy, A.N. Gleaton, L. Moon, J. Singleton, S.V. Basavaraju, C.D. Paddock); Corpus Christi Nueces County Public Health District, Corpus Christi, Texas, USA (R. Hinojosa); Methodist Transplant Institute, San Antonio (M.H. Kapturczak); Texas Department of State Health Services, Austin, Texas, USA (B. Mayes).

**Keywords:** *Rickettsia typhi*, murine typhus, flea-borne, typhus, vector-borne infections, bacteria, transplantation, Texas, United States

## Abstract

Murine typhus, a fleaborne disease caused by the bacterium *Rickettsia typhi,* is found throughout temperate and tropical regions of the world. Transmission of *R. typhi* to humans involves several species of fleas, and most infections result from direct inoculation of *R. typhi*–infected flea feces into abrasions in the skin. We describe the transmission of *R. typhi* from an organ donor in Texas, USA, to 2 kidney transplant recipients. The donor and 1 recipient died from the infection. The occurrence of *R. typhi* transmission via transplantation is a harbinger for the reemergence of murine typhus in some of the most densely populated metropolitan areas of the United States. Our findings reinforce the need to improve healthcare provider and public awareness of this life-threatening but treatable infection.

Murine or fleaborne typhus, an infection caused by the endotheliotropic bacterium *Rickettsia typhi,* occurs throughout temperate and tropical regions of the world. Murine typhus typically manifests as an undifferentiated febrile illness with severe headache, myalgia, arthralgia, and malaise (*1*–*4*) but can progress to severe, multisystem disease characterized by septic shock, pneumonia, hepatitis, acute kidney injury, myocarditis, meningoencephalitis, hemophagocytic lymphohistiocytosis, or death (*3*–*9*). The classical transmission cycle of *R. typhi* involves commensal rodents in the genus *Rattus* and Oriental rat fleas (*Xenopsylla cheopis*) (*10*). During the past several decades, investigators have identified a second transmission cycle in the United States involving cat fleas (*Ctenocephalides felis*), opossums, and feral cats (*11*–*13*). Most human infections are believed to result from inoculation of *R. typhi*–infected flea feces into abrasions in the skin and less often from inhalation or inoculation of mucous membranes with infectious feces (*10*). Murine typhus was documented once previously in a transplant recipient, albeit 5 years posttransplantation (*14*). In this article, we describe 2 transplant recipients who acquired murine typhus from organs received from a common donor. This donor resided in a region of the United States where an extraordinary resurgence of this disease has occurred after more than half a century of relative quiescence (*15*–*17*). In addition, we discuss how this event could signal broader epidemiologic implications in the 21st Century.

## Case Reports

### Transplant Recipients

**Figure 1:**
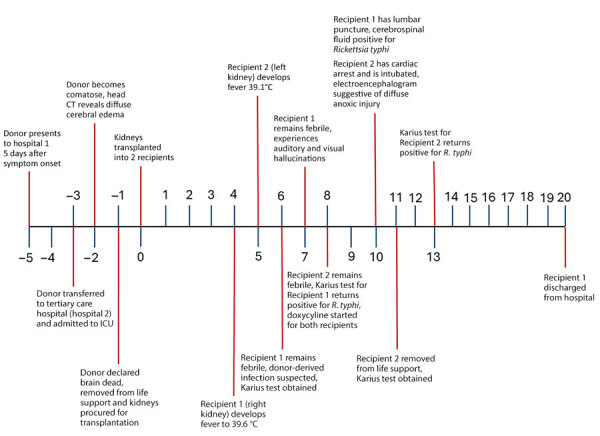
Timeline describing the transmission of *Rickettsia typhi* from an organ donor to 2 kidney transplant recipients, Texas, 2024. Day 1 of donor hospitalization is represented as −5, the day of death as −1, and the day of transplantation as 0, after which posttransplant days are listed, ending with the discharge of recipient 1 from the hospital on posttransplant day 20. CT, computed tomography; ICU, intensive care unit; Karius, https://kariusdx.com.

In October 2024, a febrile illness developed in 2 kidney recipients from a common donor several days after transplantation (Figure 1). Recipient 1, a 47-year-old man with end-stage renal disease secondary to diabetes type 2, received the right kidney and remained hospitalized because of delayed graft function. On the fourth day after transplantation, a fever to 39.6°C developed in recipient 1 (Figure 1). Laboratory tests revealed anemia, hyponatremia, and elevated creatinine (Appendix Table 1). Physical examination revealed no specific findings. We obtained blood and urine cultures and treated the patient empirically with cefepime. Micafungin was added after isolation of a *Candida* sp. from donor urine. Results of computed tomography (CT) without contrast of the chest, abdomen, and pelvis were unremarkable. The recipient’s fever persisted, and on day 6, we collected plasma for microbial cell-free (mcfDNA) metagenomic sequencing by using the Karius test (Karius, https://kariusdx.com). The next day, the patient experienced auditory and visual hallucinations, and a severe frontal headache developed. The Karius test results were returned on day 8 and reported mcfDNA of *R. typhi* in plasma at 65,818 molecules/µL (Appendix Table 1). The transplant team provided this unexpected result to the organ procurement organization (OPO), who subsequently notified the organ procurement and transplantation network (OPTN). We administered doxycycline intravenously to recipient 1. The next day, thrombocytopenia and hypoalbuminemia developed in the patient (Appendix Table 1). A head CT without contrast on day 10 was unremarkable. We performed a lumbar puncture the same day, and routine cultures of cerebrospinal fluid (CSF) were negative. We discharged the patient on the 20th day after kidney transplant.

Recipient 2, a 46-year-old woman with end-stage renal disease secondary to diabetes type 2 and hypertension, received the left kidney and remained hospitalized for delayed graft function. On day 5 after transplantation, a fever to 39.3°C developed in recipient 2 (Figure 1). Laboratory tests revealed anemia, hyponatremia, and elevated levels of aspartate aminotransferase, alkaline phosphatase, and creatinine (Appendix Table 1). Physical examination revealed no specific findings. We obtained blood and urine cultures, and the patient was treated empirically with cefepime. Her fever persisted, and on day 8 a severe diffuse headache developed. We began intravenous doxycycline for recipient 2 on the same day that the Karius test result for *R. typhi* returned for recipient 1. Recipient 2 remained febrile, and on day 9, she exhibited echolalia and was transferred to an intensive care unit. The next day she experienced cardiac arrest and was intubated. An electroencephalogram showed diffuse suppression suggestive of diffuse anoxic injury. Magnetic resonance imaging revealed extensive global brain injury. A Karius test was obtained on the same day and detected a nonquantifiable level of *R. typhi* DNA (Appendix Table 1). Her family made the decision to withdraw life support, and recipient 2 died 11 days after transplant.

### Organ Donor

**Figure 2:**
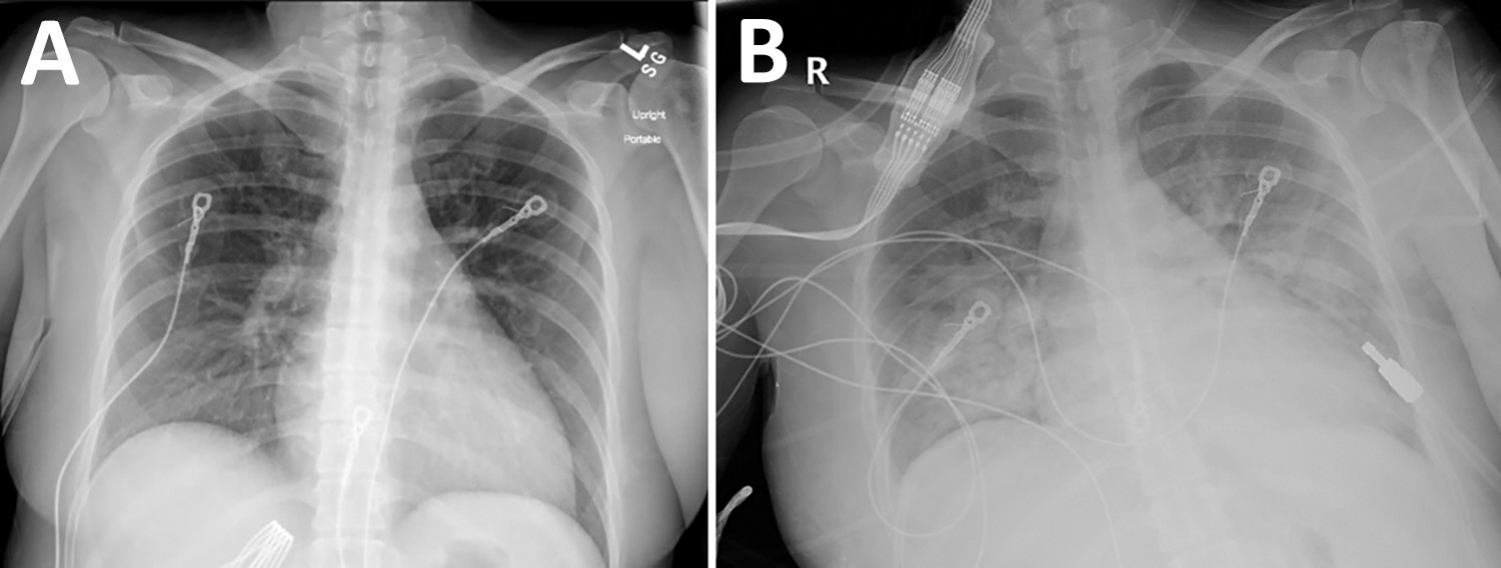
Chest radiographs from an organ donor with murine typhus, from a study describing the transmission of *Rickettsia typhi* from an organ donor to 2 kidney transplant recipients, Texas, 2024. A) Chest radiograph obtained at hospital admission, 5 days after symptom onset, with no specific abnormalities noted. B) Chest radiograph obtained on hospital day 3, revealing extensive alveolar opacities predominantly in the middle and lower lung fields.

Five days before her death, a 34-year-old woman was seen at an emergency department in south Texas with complaints of 5 days of fever, lower extremity weakness, cough, dyspnea, headache, and syncope. She had a history of uncontrolled thyroid endocrinopathy and noncompliance with thyroid medications. Physical examination revealed a jaundiced, toxic-appearing woman with a temperature of 36.1°C, heart rate of 114 beats/min, and respiratory rate of 20/min. No rash was found. Laboratory abnormalities included leukocytosis; thrombocytopenia; anemia; hyperbilirubinemia; multiple hepatic enzyme abnormalities; elevated free triiodothyronine, free thyroxine, and total thyroxine; and low thyroid-stimulating hormone (Appendix Table 1). A chest radiograph revealed no abnormalities (Figure 2, panel A). Doppler ultrasound of her thyroid gland revealed enlargement and marked hypervascularity. She was diagnosed with thyrotoxicosis and was started on methimazole, propranolol, cholestyramine, and hydrocortisone. Uncontrolled tachycardia (133 beats/min) and worsening dyspnea developed on her third hospital day. A chest radiograph revealed extensive, bilateral, alveolar opacities most prominent in the middle and lower lungs (Figure 2, panel B). She required mechanical ventilation and was transferred to the intensive care unit of a tertiary care center. A chest radiograph revealed extensive alveolar opacities in her middle and lower lungs. Within 24 hours, she became comatose, and head CT revealed diffuse cerebral edema with obliteration of the basal cisterns. A nuclear medicine perfusion scan showed absent cerebral circulation, and she was declared brain dead on the fifth hospital day and withdrawn from life support. After donor eligibility screening and standard tests performed by the OPO, her kidneys were procured for transplantation.

## Methods

### Clinical and Epidemiologic Review

In the United States, all unexpected, suspected transplant-transmitted diseases must be reported by OPOs or transplant centers to the OPTN for investigation by the ad hoc disease transmission advisory committee. Cases involving pathogens of special interest (https://optn.transplant.hrsa.gov/media/yyhnrkar/special_pathogens_list.pdf) are reported to the Centers for Disease Control and Prevention (CDC) for investigation. CDC was notified by the OPTN disease transmission advisory committee when mcfDNA of *R. typhi* was detected in the plasma of recipient 1. State and local public health officials and the OPO identified residual tissue and body fluid specimens from the organ donor and recipients for confirmatory testing at CDC. Donor and recipient medical records were reviewed by local health officials and by the clinical team. After confirmation of murine typhus in the donor, local public health officials conducted an environmental assessment of the donor’s residence in south Texas. This activity was reviewed by CDC and conducted consistent with applicable federal law and CDC policy.

### Laboratory Methods

We extracted DNA from residual blood, plasma, serum, CSF, or tissue specimens from the donor and recipient 1. We tested the extracted DNA by using a real-time PCR that amplified a 111-bp segment of the 23S rRNA gene of all *Rickettsia* sp. (*18*) and by using real-time PCRs that amplify 146-bp or 197-bp segments of intergenic regions from the genome of *R. typhi.* The primers and probes and for each target are provided (Appendix Table 2). Threshold cycle (Ct) values <40 were considered positive. A residual serum specimen from the donor was tested for *R. typhi* IgG by an indirect immunofluorescence assay (*19*). We considered a titer >128 positive. We evaluated formalin-fixed, paraffin-embedded biopsies of the right and left kidney allografts obtained before transplantation by an immunohistochemical (IHC) stain to detect antigens of typhus group *Rickettsia* sp. (*20*).

## Results

**Figure 3:**
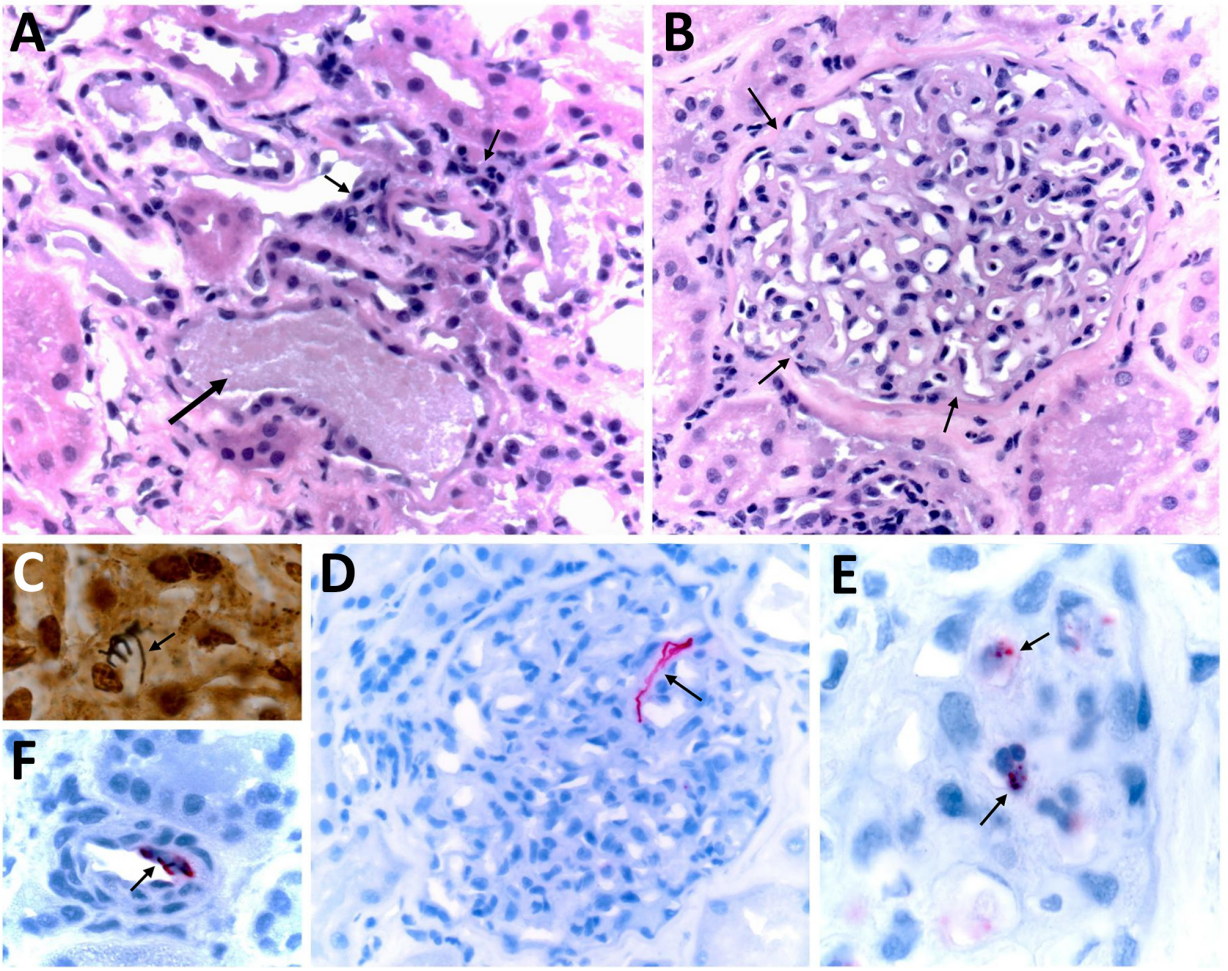
Histopathological and immunohistochemical features of formalin-fixed, paraffin-embedded biopsy specimens collected from the right and left kidney allografts procured from a donor who died of murine typhus, from a study describing the transmission of *Rickettsia typhi* from an organ donor to 2 kidney transplant recipients, Texas, 2024. A) When stained with hematoxylin and eosin, both allografts showed multifocal, interstitial, and predominantly mononuclear peritubular infiltrates, associated with focal endarteritis (thin arrows), features of acute tubular injury, including epithelial attenuation with loss of apical cytoplasm, and pigmented casts (large arrow). B) Glomeruli displayed moderate mesangial hypercellularity and tuft adhesions to the Bowman’s capsules (arrows) and intracapillary and mesangial phagocytic foam-cells. C) Short chains comprising small, rod-shaped bacteria were revealed in glomerular capillaries by the Warthin–Starry silver impregnation staining technique. D–F) An immunohistochemical stain for typhus-group rickettsiae revealed intact bacteria within endothelial cells of inflamed small vessels and vascular spaces of mesangial capillaries (arrows) and phagocytized bacterial antigens in the cytoplasm of glomerular foam-cells (arrows). Original magnifications ×400 (A, B, and E) and ×1,000 (C, D, and F).

We amplified DNA of *R. typhi* from residual donor samples collected at the time of organ procurement that had mean Ct values of 30.52 (plasma), 30.12 (serum), 32.35 (whole blood), 32.29 (lymph node), and 28.16 (spleen). Antigens of *R. typhi* were detected by IHC in donor lymph node and spleen, and *R. typhi* IgG at a titer of 512 were detected by immunofluorescence assay in donor serum samples obtained on the day of her death. We amplified *R. typhi* DNA at a Ct of 38.86 from residual CSF collected from recipient 1 on posttransplant day 11 (Appendix Table 1). Histopathologic review of the pretransplant allograft biopsies from each transplanted kidney revealed multifocal and predominantly mononuclear inflammatory infiltrates in interstitial peritubular stroma, focal endarteritis, acute tubular injury, and pigmented casts in tubular lumina (Figure 3, panel A). Some glomeruli showed features of focal segmental glomerulosclerosis, including mesangial hypercellularity and tuft adhesions to the Bowman’s capsules (Figure 3, panel B). Short chains comprising small, rod-shaped bacteria were identified by the Warthin–Starry silver impregnation staining method (*21*) in a few glomeruli (Figure 3, panel C). Antigens of *R. typhi* were detected by IHC stain within endothelial cells of small interstitial blood vessels and in the mesangium of several glomeruli (Figures 3, panels D–F).

An epidemiologic assessment of the donor’s residence revealed tall grass and abundant loose garbage, wooden planks, and other materials scattered around the yard. An interview conducted with the donor’s partner indicated that she had frequent exposures to ≈10 flea-infested stray cats that lived on the property.

## Discussion

**Figure 4:**
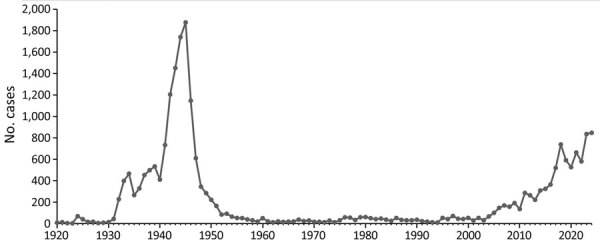
Annual case counts of murine typhus in Texas, USA, during 1920–2024.

This identification of murine typhus acquired by organ transplantation provides further evidence for the resurgence of this disease in the United States, particularly south-central Texas (*3*,*4*,*17*,*19*,*22*) and southern California (*9*,*23*). During 1931–1946, ≈42,000 cases of murine typhus were reported in the United States, predominantly from Alabama, Florida, Georgia, Louisiana, Mississippi, South Carolina, and Texas (*15*). Estimates of unrecorded cases outnumbered recorded cases by ≈4 to 1 (*10*). From the late 1940s through the early 1950s, coordinated campaigns by local, state, and national public health agencies across the southeastern United States effectively controlled the disease by intensive rodent elimination programs and widespread application of DDT around rodent harborages. National case counts of murine typhus plummeted during the late 1940s, and from the late 1950s through the late 1980s, <100 cases of murine typhus were recorded annually in the United States (*15*,*16*). Murine typhus was removed from the list of nationally notifiable diseases in 1987 (*16*). However, states such as California and Texas, where the disease remained a reportable condition, have recorded huge increases in annual case counts during the past decade (*9*,*16*,*17*,*23*). For example, the cumulative number of cases in Texas during 2014–2024 was 4.6 times greater than during 2003–2013 (https://www.dshs.texas.gov/notifiable-conditions/zoonosis-control/zoonosis-control-diseases-and-conditions/flea-borne-typhus), a trend reminiscent of the explosive rise of murine typhus in Texas almost a century ago (Figure 4)

Murine typhus can progress to severe or life-threatening disease, as evidenced by contemporary surveillance summaries, from which ≈60%–83% of patients are hospitalized (*7*,*24*); among that cohort, 5%–30% are admitted to an intensive care unit (*3*,*4*,*19*,*22*,*24*.*25*), often at admission to a hospital (*4*,*8*,*26*–*29*). In this report, the donor died 9 days after the onset of otherwise undifferentiated febrile illness attributed to thyrotoxicosis. The diagnosis of murine typhus is commonly missed in patients seeking care (*1*,*4*,*29*,*30*), even for those who are critically ill (*5*–*9*). This fact reflects the clinical dilemma posed by murine typhus, even in locations such as south Texas (*4*,*22*,*25*,*31*,*32*), where the disease has remained endemic for >100 years (*33*–*35*). In this article, an otherwise unexpected diagnosis of murine typhus in each kidney recipient was confirmed by metagenomic sequencing of mcfDNA, similar to multiple recent reports from California and Texas that have achieved diagnoses by using this technology (*6*,*9*,*29*–*31*).

Early clinical descriptions of murine typhus comment on the frequency of a short, hacking cough in 54% to >90% of patients (*1*,*2*,*36*) that for many “was likely to be unnoticed until attention was directed toward it” (*36*). In this report, the donor had multiple complaints that included cough and dyspnea. Either or both of those clinical findings are documented in 35%–59% of contemporary United States patient series (*1*–*4*,*22*–*23**, **25*), but they are infrequently identified as notable clinical findings. Radiographic evidence of more advanced pulmonary disease, including pneumonitis or pulmonary infiltrates, is described infrequently in most patient series; however, a recent analysis of 621 patients from 9 worldwide cohort studies identified chest radiograph abnormalities in 104 (16.7%) patients (*37*). *R. typhi* can remain infectious in desiccated flea feces at ambient temperatures for 9–15 months (*38*). That observation, evaluated in conjunction with the frequency of early respiratory findings (*1*,*2*,*36*,*39*), suggests that infections acquired directly from inhalation of desiccated flea feces could be more common than considered previously.

Each patient in this series demonstrated central nervous system involvement, also consistent with historical and contemporaneous patient series that identify photophobia in 12%–17% of patients, eye pain in 11%–14% of patients, nuchal rigidity in 6%–22% of patients, and stupor and delirium in 16%–17% of patients (*1*,*2*,*4*,*23*). Two of the patients in this series died from infection with *R. typhi.* During the preantimicrobial era, the case-fatality rate of murine typhus in the southeastern United States was estimated at 3.8%–4.3% (*36*,*39*). Contemporary case-fatality rates of murine typhus in the United States are <1% (*33*,*40*), although those estimates are confounded by an unknown number of otherwise undiagnosed survivors and, perhaps more important, an unknown number of undiagnosed deaths. The recent identification of 3 fatal cases of murine typhus in Los Angeles County, California, in a single year exemplifies the lethal potential of this disease (*9*). In that context, the historical perception of murine typhus as a relatively mild rickettsiosis that typically runs an otherwise benign and self-limited clinical course (*41*) requires reexamination.

Arthropodborne pathogens are increasingly recognized as infectious agents capable of transmission via organ transplantation (*42*–*44*). Not all OPOs collect standardized data for known or potential recent arthropod and animal exposures; nonetheless, routine acquisition of those data could provide early clues to OPOs, transplant centers, and clinicians to consider vectorborne diseases in ill recipients during the early posttransplant period, particularly among recipients who develop otherwise unexplained multisystem disease. Such information could thereby guide and expedite empiric therapy and posttransplantation evaluation for specific vectorborne pathogens.

The public health triumph that almost eliminated murine typhus in the United States during the second half of the 20th Century paradoxically eroded general medical awareness of the disease. For example, the initial diagnosis for 44% of 180 patients was murine typhus in a series from Louisiana (*1*) in 1945, near the historical zenith of annual case counts in the United States (*10*,*15*). Several decades later, only 11% of 345 cases described from Texas during 1980–1987 received an initial diagnosis of murine typhus (*25*). More recently, murine typhus diagnosis was not considered initially for any of 10 acutely ill patients referred to a tertiary care center in Texas during 2017–2020 (*29*), or for any of 23 children from Texas when first evaluated as outpatients by primary care physicians and who later required hospitalization (*3*).

The environmental and social factors contributing to the resurgence of murine typhus in California (*9*,*23*) and Texas (*3*,*4*,*19*,*22*) are not identical to the conditions responsible for the explosive emergence of murine typhus in the United States during the 1930s and 1940s (*45*). The contemporary ecology and epidemiology of murine typhus in the United States now includes multiple newly recognized species of urban wildlife, including opossums and feral cats (*11*–*13*,*16*,*17*,*46*), and a second, highly ubiquitous vector flea species (*47*,*48*). Increasing numbers of homeless persons in the United States introduce a cohort of persons at increased risk for exposure to the suite of vertebrate reservoirs and vectors that perpetuate the transmission of *R. typhi* in urban settings (*9*,*23*). Those conditions are not restricted to California and Texas and could therefore contribute to additional foci of reemergence in other states affected historically by this disease (*8*,*15*,*16*,*45*,*46*). Efforts are needed to improve healthcare provider and public awareness of this life-threatening and treatable infection that was largely forgotten but never gone (*49*,*50*).

Appendix Additional information about organ donor transmission of *Rickettsia typhi* to kidney transplant recipients, Texas, USA, 2024
